# Biosynthetic Functional Gene Analysis of *Bis*-Indole Metabolites from 25D7, a Clone Derived from a Deep-Sea Sediment Metagenomic Library

**DOI:** 10.3390/md14060107

**Published:** 2016-06-01

**Authors:** Xia Yan, Xi-Xiang Tang, Dan Qin, Zhi-Wei Yi, Mei-Juan Fang, Zhen Wu, Ying-Kun Qiu

**Affiliations:** 1 School of Pharmaceutical Sciences, Xiamen University, South Xiang-An Road, Xiamen 361102, China; yanxia1201@126.com (X.Y.); fangmj@xmu.edu.cn (M.-J.F.); 2 Key Laboratory of Marine Biogenetic Resources, Third Institute of Oceanography State Oceanic Administration, Xiamen 361005, China; tangxixiang@tio.org.cn (X.-X.T.); yizhiwei@tio.org.cn (Z.-W.Y.); 3 South China Sea Bio-Resource Exploitation and Utilization Collaborative Innovation Center, Xiamen 361005, China; 4 Key Laboratory of Urban Pollutant Conversion, Institute of Urbane Environment, Chinese Academy of Sciences, Xiamen 361021, China; dqin@iue.ac.cn

**Keywords:** deep-sea sediment, metagenomic clone, secondary metabolites, diindole derivatives, biosynthetic functional genes, cytotoxicity

## Abstract

This work investigated the metabolites and their biosynthetic functional hydroxylase genes of the deep-sea sediment metagenomic clone 25D7. 5-Bromoindole was added to the 25D7 clone derived *Escherichia coli* fermentation broth. The new-generated metabolites and their biosynthetic byproducts were located through LC-MS, in which the isotope peaks of brominated products emerged. Two new brominated *bis*-indole metabolites, 5-bromometagenediindole B (**1**), and 5-bromometagenediindole C (**2**) were separated under the guidance of LC-MS. Their structures were elucidated on the basis of 1D and 2D NMR spectra (COSY, HSQC, and HMBC). The biosynthetic functional genes of the two new compounds were revealed through LC-MS and transposon mutagenesis analysis. 5-Bromometagenediindole B (**1**) also demonstrated moderately cytotoxic activity against MCF7, B16, CNE2, Bel7402, and HT1080 tumor cell lines *in vitro*.

## 1. Introduction

Marine microorganisms exhibit unique metabolic properties because of the particularity of marine environments; as a result, many novel chemical structures are very complex and diverse. Many antitumor, antibacterial, and anti-inflammatory bioactive substances have been recognized and investigated. Deep marine subsurface sediments are extensive microbial habitats on Earth; these sediments contain numerous undeveloped functional gene clusters to encode the biosynthesis of natural products. However, numerous microorganisms cannot be cultivated [1,2].

Metagenomics, which involves culture-independent methods to access the collective genomes of natural bacterial populations, is applied to investigate secondary metabolites produced by large collections of bacteria that exist in the environment but remain recalcitrant to culturing [3,4]. In metagenomics, microbial DNA is directly extracted from environmental samples and cloned into appropriate educable host cells [5,6,7]; as such, metagenomics has been employed to utilize microbial genes from extreme environments, which are associated with the production of bioactive small molecules. We previously isolated a new compound and found a potent analgesic activity on fatty acid amide hydrolase (FAAH) and monoacylglycerol lipase (MGL) from the deep-sea sediment metagenomic clone 11F6 [8]. In further studies on deep-sea sediment eDNA libraries, two new indole alkaloids were isolated from a cytotoxic clone coded QD15 [9].

In addition to new metabolites from metagenomic libraries, their biosynthetic pathways should be elucidated because these pathways link genetic materials to biocatalytic enzyme functions and bioactive metabolites. The mechanisms by which metabolites are produced under ambient conditions can be determined by exploring biosynthesis-related relevant precursors and enzymes. On the basis of biosynthesis patterns, we can add the same or similar precursors or related enzyme inhibitors to obtain new structures or similar secondary metabolites. Our study investigated the metabolites of a deep-sea sediment metagenomic clone 25D7 with functional gene clusters of hydroxylase; this study also determined the proposed biosynthetic pathways of the metabolites. 5-Bromoindole was added to the culture medium to trace the secondary metabolites produced by the functional genes and to guide separation. As a result, two new brominated *bis*-indole metabolites, namely, 5-bromometagenediindole B (**1**) and 5-bromometagenediindole C (**2**), were isolated through LC-MS. The biosynthetic functional genes of the two new compounds were revealed through HPLC-MS and transposon mutagenesis analysis. 5-Bromometagenediindole B (**1**) demonstrated moderately cytotoxic activity against MCF7, B16, CNE2, Bel7402, and HT1080 tumor cell lines *in vitro*. By contrast, 5-bromometagenediindole C (**2**) did not elicit cytotoxic effects on the tumor cell lines.

## 2. Results and Discussion

### 2.1. Bromo-Substituted Substrate Fermentation and LC-MS-Guided Separation

**Figure 1:**
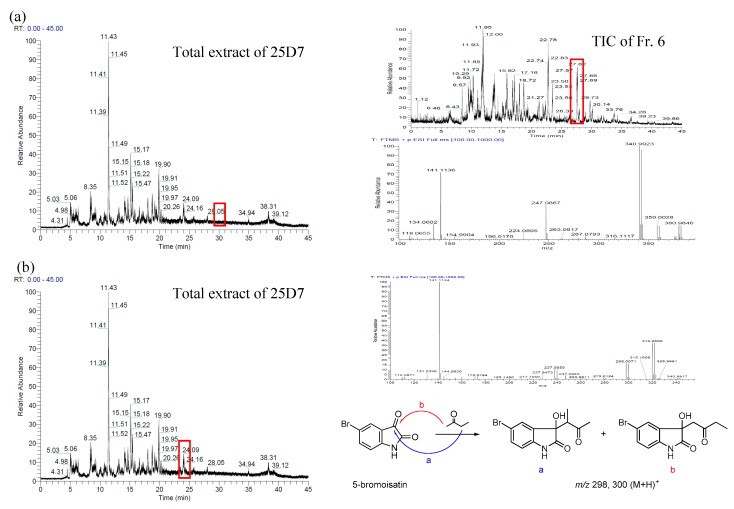
Brominated metabolites in the total extract and separated fractions determined through HPLC-MS. (**a**) Brominated metabolites found in the total ion chromatograms (TICs) of the total extract of 25D7 and subfraction Fr. 6; (**b**) Key brominated by products (butanone addition form) of the target metabolites found in the TIC of the total extract of 25D7 at *t*_R_ 24.09 min.

Our previous studies on the metabolites of metagenomic clones indicated that clones with exogenous genes can use indole generated by *Escherichia coli* as a material to synthesize new structures. In our present study, 5-bromoindole was added to the culture medium of 25D7 to obtain special metabolites produced by heterologously expressed functional genes and to verify their proposed biosynthetic pathways. The newly generated metabolites and their biosynthetic byproducts could be easily located through LC-MS. The isotope ion peak pair of [M]^+^/[M + 2]^+^ (1:1) introduced by the bromo atom may emerge. The metabolites were also separated from metagenomic clone fermentation broth under the guidance of LC-MS. The total ion chromatogram of the crude extracts of 25D7 (Figure 1a) revealed the quasi-molecular ion peaks of brominated metabolites at a retention time (*t*_R_) of 28.96 min: 340/342 [(M–OH + H)^+^], 359/361 [(M + H)^+^], and 380/382 [(M + Na)^+^]. The total extract was placed in a silica gel column and an ODS column for further separation. Each separated fraction was analyzed through LC-MS to specifically locate the target fraction with the brominated metabolites. Two new brominated *bis*-indole metabolites, namely, 5-bromometagenediindole B (**1**) and 5-bromometagenediindole C (**2**), were separated. The key by products of butanone addition, which involves nucleophilic addition when extraction is performed with butanone, were detected in the HPLC-MS spectra at *t*_R_ of 24.09 min with *m/z* 298/300 [(M + H)^+^] and 319/321 [(M + Na)^+^] (Figure 1b). These by products were then isolated. The related biosynthetic products and intermediate hydroxylated indoles with or without 5-bromo substituent were obtained or detected through HPLC-MS (Supplementary Materials).

### 2.2. Structural Identification of the Two New Bromodiindoles

5-Bromometagenediindole B (**1**) was isolated as a colorless crystal with a molecular formula of C_16_H_11_N_2_O_3_Br on the basis of HRESIMS ([M + Na]^+^ at *m/z* 380.9841, calcd. for C_16_H_11_N_2_O_3_BrNa) coupled with 1D NMR spectroscopic data. The IR spectrum of **1** exhibited absorption bands at 3378 cm^−1^ (NH and/or OH) and 1693 (C=O) cm^−1^. The UV spectrum of **1** in methanol exhibited maximum absorbance at 224 and 262 nm. In the *sp*^2^ region of ^1^H NMR (Table 1), a set of three-spin AMX proton system signals at δ_H_ 7.07 (1H, overlapped, H-4), 7.40 (1H, dd, *J =* 8.3, 1.5 Hz, H-6), and 6.90 (1H, d, *J =* 8.5 Hz, H-7). A set of two-spin AX proton system signals at δ_H_ 7.06 (1H, d, *J =* 8.1 Hz, H-5′), 6.51 (1H, d, *J =* 8.1 Hz, H-6′) was also shown. Considering the biotransformation of 5-bromoindole substrates and the two labile proton at δ_H_ 10.95 (1H, s) and 10.58 (1H, br. s) in the low field of the ^1^H NMR of **1**, we could propose the presence of two indole moieties. The ^1^H–^1^H COSY correlations of H-6/H-4,7 and H-5′/H-6′ indicated that the AMX and AX spin systems were attributed to 2,3,5-trisubstituted indole and 4′,7′-dissubstituted indole moieties. In the ^1^H-NMR spectrum of **1**, the aromatic hydroxyl signal at δ_H_ 9.65 (1H, br. s, –OH) could be attributed to 7′-OH, as shown by the high field chemical shift of H-6′ at δ_H_ 6.51. Another hydroxyl signal from alcohol was presented at δ_H_ 6.58 (1H, br. s, –OH). This finding revealed the existence of *sp*^3^ carbon at one of the five-membered rings in the two indole moieties. The ^13^C NMR spectra illustrated the differences in the five-membered rings. In addition to benzene carbons, two additional *sp*^2^ carbon signals at δ_C_ 126.3 (s, C-7′a) and 126.6 (s, C-3′a) were attributed to one of the indole moieties. A ketone carbon at δ_C_ 178.8 (s, C-2) and an *sp*^3^ quaternary carbon at δ_C_ (s, C-3) belonged to another indole moiety.

**Figure 2:**
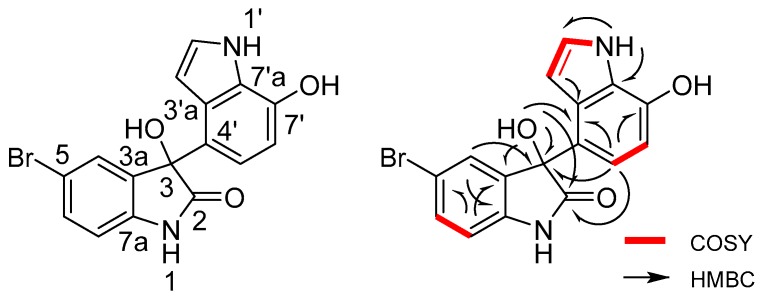
Structure and key ^1^H–^1^H COSY and HMBC correlations of 5-bromometagenediindole B (**1**).

In the HMBC spectrum of **1**, the correlations of 3-OH/C-2, 3, and 3a explained the reduction and hydroxyl substitution of C-3 in the indole moiety. The observed ^1^H–^1^H COSY correlations between H-5′ and H-6′ and the HMBC correlation between H-5′ and 6′/C-7′ confirmed that C-7′ of the other indole ring was substituted by another hydroxyl. The HMBC signals between H-5′/C-2, 3 and 3-OH/C-4′ indicated that C-4′ was attached to C-3. The molecular framework was established on the basis of the HMBC correlations (Figure 2). The structure of **1** is 5-bromo-3,7′-dihydroxy-1,3-dihydro-1′*H*,2*H*-3,4′-biindol-2-one, named 5-bromometagenediindole B. It is a newly isolated compound produced by the deep-sea sediment metagenomic clone 25D7 through bromo-substrate addition. The optical rotation of **1** is close to zero, and this finding indicates that **1** is a racemate. Similar indole-based compounds have also been found in different metagenomic libraries. For example, metagenetriindole A and metagenediindole A with moderately cytotoxic activity against CNE2, Bel7402, and HT1080 tumor cell lines *in vitro* has been isolated from the deep-sea sediment metagenomic clone QD15 [9]. Abe *et al.* constructed a metagenomic library from the marine sponge *Halichondria okadai* and then isolated a novel compound named halichrome A [10]. Yao Wang *et al.* obtained three new indole alkaloids, namely, shewanellines A, B, and C, from *Shewanella piezotolerans* WP3 collected in deep-sea sediments; shewanelline B can significantly inhibit the growth of human tumor cell line HL-60 [11]. However, this is the first report on the isolation of *bis*-indole with a phenol-substituted indole ring from a metagenomic clone.

**Figure 3:**
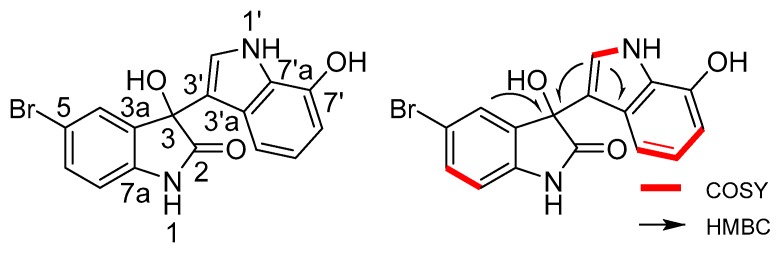
Structure and key ^1^H–^1^H COSY and HMBC correlations of 5-bromometagenediindole C (**2**).

5-Bromometagenediindole C (**2**) was isolated as a colorless crystal with a molecular formula of C_16_H_11_N_2_O_3_Br on the basis of HRESIMS ([M + Na]^+^ at *m*/*z* 380.9845, calcd. for C_16_H_11_N_2_O_3_BrNa), which is the structural isomer of **1**. The 5-bromo-substituted indole ring moiety is identical to that in **1** because the corresponding ^1^H and ^13^C NMR data could be found in compound **2**. In the ^1^H NMR spectrum of compound **2** (Table 2), a set of three-spin proton ABX system signals emerged at δ_H_ 6.69 (1H, d, *J =* 7.5 Hz, H-4′), 6.66 (1H, t, *J =* 7.5 Hz, H-5′), and 6.44 (1H, d, *J =* 7.4 Hz, H-6′). In the ^1^H-^1^H COSY spectra, the correlation between H-4′/H-5′/H-6′ and H-1′/H-2′ indicated the presence of 5′-H unsubstituted 7′-OH indole moiety, which differed from that in **1**. The linkage of the two indole moieties was revealed by the HMBC correlations between H-2′/C-3 and H-4/C-3. The structure of **2** is 5-bromo-3,7′-dihydroxy-1,3-dihydro-1′*H*,2*H*-3,3′-biindol-2-one, named 5-bromometagenediindole C. The structure and key ^1^H–^1^H COSY and HMBC correlations are shown in Figure 3. Similar bromo-diindole compounds without hydroxyl at position 7′ were synthesised by Chauhan *et al.* with 5-bromoistain and indole. The C-7′ ^13^C NMR signal of the reported compound was up-field shifted compared with that of **2** [12].

### 2.3. Biosynthetic Functional Gene Analysis of Bromo-Diindoles

Gene expression-induced changes in metabolites should be elucidated to clarify the role of functional genes in metabolite production. Hence, biosynthetic pathways and associations between functional genes and metabolites should be investigated.

**Figure 4:**
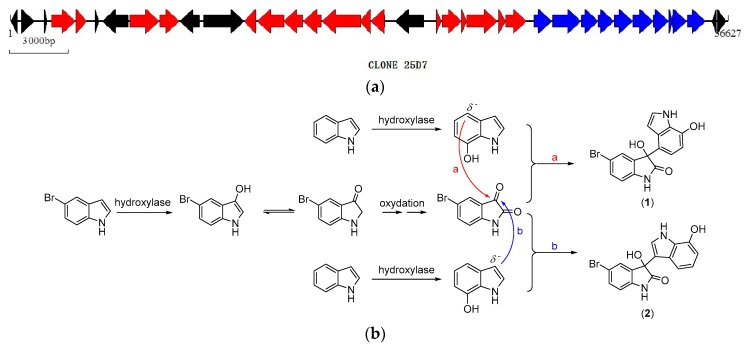
Functional gene clusters of 25D7 (**a**) and the proposed biosynthetic pathways of 5-bromodiindoles (**b**).

25D7 contains a 36,627 bp insertion sequence with 37 predicted CDSs (GenBank accession number: KU883232). The potential functional gene clusters of 25D7 with six phenol hydroxylase subunit genes are shown in Figure 4a and Table 3. The proposed biosynthetic pathways of 5-bromodiindoles were illustrated as Figure 4b. The expression of hydroxylase functional genes produced hydroxylase, which oxidizes C-2 and C-3 of the indole ring and produce 5-bromoisatin, which is a key by product of this reaction [13,14,15]. The C-3 carbonyl group of 5-bromoisatin is a positively charged electrophilic group, which can be easily attacked by a nucleophilic group. Hydroxylase also induces the hydroxylation of indole produced by *E. coli* at position 7. Position 3 and 4 of 7-hydroxy indole is a negatively charged nucleophilic group. 5-Bromometagenediindole B (**1**) is produced when nucleophilic addition occurs at C-4 of 7-hydroxy indole. 5-Bromometagenediindole C (**2**) is generated when nucleophilic addition occurs at C-3. The optical data of the new compounds are close to zero, indicating the non-stereoselectivity of this addition reaction step.

Considering the proposed biosynthetic scheme, debromo derivatives should exist in the extract. Such derivatives were detected. For example, the debromo metagenediindole B/C were found in the HPLC-MS spectra at Rt 20.22 min with *m/z* 281 [(M + H)^+^] and 303 [(M + Na)^+^]. Indigo and indirubin, including 5-bromo substituted derivatives, were also produced by catalytic hydroxylation followed by auto-oxidation. Other by-products, such as 5-hydroxyl indole and 5-bromo-6-hydroxyl indole, were isolated and their spectra data were given in Supplementary Materials.

**Figure 5:**
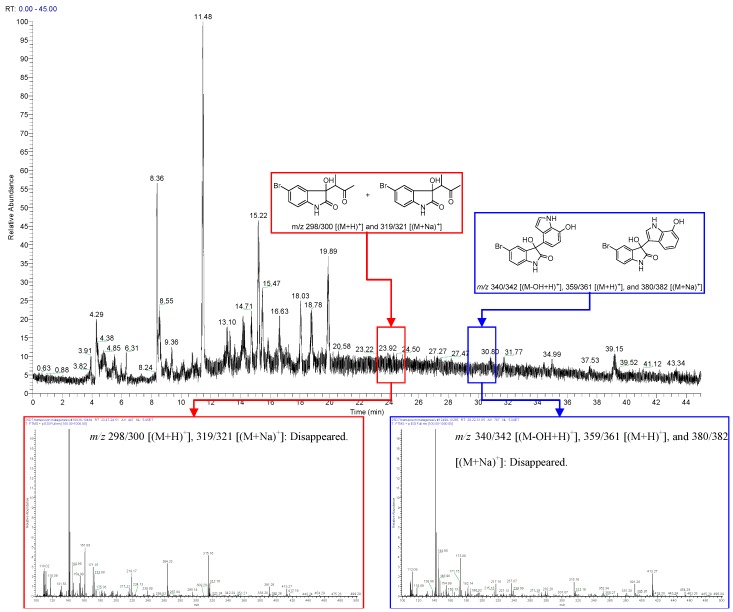
HR-ESI-MS analysis of the transposon mutagenesis of 25D7.

*In vitro* transposon mutagenesis was conducted and HR-ESI-MS was performed to locate the genes responsible for bromo-diindole production. 25D7 appeared brown in the culture medium, and hundreds of colorless mutants in LB-tyrosine plates were obtained. The sequencing of the 30 randomly selected mutants revealed that their sequences were disrupted; this result indicated the high identity of the genes to bacterial phenol hydroxylase subunit genes (CDS 20–25). The HR-ESI-MS data of the mutants further illustrated that the two new bromo-diindoles and 5-bromoisatin were missing (Figure 5).

### 2.4. Cytotoxic Activity of 5-Bromodiindoles

The cytotoxic activities of the two new indole alkaloids were evaluated using MCF7, B16, CNE2, Bel7402, and HT1080 cell lines via the CCK-8 method [9]. The results revealed that 5-bromometagenediindole B (**1**) demonstrated moderately cytotoxic activity against the five tumor cell lines *in vitro*, with IC_50_ of 20.34, 16.60, 32.54, 27.48, and 15.26 μg·mL^−1^ respectively. By contrast, 5-bromometagenediindole C (**2**) did not elicit cytotoxic effects on the tumor cell lines.

## 3. Experimental Section

### 3.1. General Experimental Procedures

LC-MS and HR-ESI-MS analyses were performed on a Dionex Ultimate 3000 UHPLC system coupled with a Thermo Q-Exactive Orbitrap mass spectrometer (Thermo Fisher Scientific Corporation, Waltham, MA, USA) equipped with an electrospray ionization source (ESI) and an analytical Cosmosil ODS column (250 mm × 4.6 mm i.d., 5 μm; Cosmosil, Nakalai Tesque Co., Ltd., Kyoto, Japan). Preparative HPLC was conducted in a Varian binary gradient LC system (Varian Inc., Corporate, Santa Clara, CA, USA) containing two solvent delivery modules (PrepStar 218), a photodiode array detector (ProStar 335), and a fraction collector (ProStar 704) by using a preparative Cosmosil ODS column (250 mm × 20.0 mm i.d., 5 μm, Cosmosil, Nakalai Tesque Co., Ltd., Kyoto, Japan). UV spectra were obtained using a Shimadzu UV-260 spectrometer (Shimadzu Corporation, Tokyo, Japan). IR spectra were determined by using a Perkin-Elmer 683 infrared spectrometer (PerkinElmer, Inc., Waltham, MA, USA) in KBr pellets. Optical rotations were performed by using a JASCO P-200 polarimeter (JASCO Corporation, Tokyo, Japan) with a 5 cm cell. NMR spectra were obtained using a Brucker Avance III 600 FT NMR spectrometer (Bruker Corporation, Billerica, MA, USA), with TMS as an internal standard. Column chromatography was performed on silica gel (Yantai Chemical Industry Research Institute, Yantai, China) and Cosmosil 75 C_18_-OPN (75 μm, Nakalai Tesque Co., Ltd., Kyoto, Japan).

### 3.2. Fermentation

Sediment samples for DNA extraction were collected from the subsurface sediments at water depths of 3006 m (102.612575° E, 2.022449° N) in Southwestern Indian Ocean by the Third Institute of Oceanography of China. The samples were maintained at 4 °C before processing. The sediment samples were pre-cultured in 2216E medium for 3 days. Then, the DNA of the enrichment product was extracted and purified. The size-separated DNAs of 30–40 kb were pooled and end-repaired to blunt end and cloned into fosmid vector. The ligation mixture was packed; the packaged DNA was transformed into *E. coli*, and a library of 3500 clones was generated. Cytotoxic activity was then screened. The brown pigment-producing 25D7 was selected and characterized. 25D7 was incubated in a 60 L fermentation tank supplemented with 30 μg/mL 5-bromoindole and 0.01% inducer and fermented for 18 h at 37 °C, 200 rpm, and pH 7.0. The supernatant from the fermentation broth was collected by using a continuous flow centrifuge at 60 L/min. A voucher specimen (25D7) has been deposited at the Third Institute of Oceanography, State Oceanic Administration of China.

### 3.3. Extraction and Isolation

The fermentation broth (60 L) was extracted thrice with butanone (*v/v* 1:1). The butanone phase was evaporated at a reduced pressure to produce the total extract (9.4 g). The total crude extract was analyzed using an analytical Cosmosil ODS-MS column (250 mm × 4.6 mm i.d., 5 μm, Nakalai Tesque Co., Ltd., Kyoto, Japan). The mobile phase used was acetonitrile (A) and water (B) in a linear gradient mode, as follows: A from 5% to 100% and B from 95% to 0% between 0 and 40 min. The flow rate of the mobile phase was 0.4 mL·min^−1^ and the effluents were monitored by a HR-ESI-MS.

The total extract was subjected to liquid chromatography on silica gel by using CHCl_3_-MeOH as an eluent at gradient elution ratios to produce seven fractions (Fr. 1 to 7). Each fraction was analyzed with HPLC-MS to fine metabolites containing a bromo atom. In Figure 1b, quasi-molecular ion peaks of brominated products appeared in Fr. 6 at a retention time of 27.76 min. Fr. 6 (0.86 g) was subjected to ODS chromatography eluted with MeOH-H_2_O (10:90 to 100:0) to yield a subfraction Fr. 6.5. Fr. 6.5 (136.3 mg) was purified through preparative HPLC by using a C18 column (acetonitrile–H_2_O, 30:70 to 35:65) to obtain **1** (24 mg) and **2** (2.5 mg).

Metagenediindole B (**1**): colorless crystal; ${\left[\alpha \right]}_{D}^{29}$ 0° (*c* = 0.1, MeOH); IR (KBr) (ν_max_): 3378, 1693 cm^−1^. UV (MeOH) λ_max_ (logε): 262 (4.03) and 224 (3.59) nm. ^13^C NMR (125 MHz, DMSO-*d*_6_) and ^1^H NMR (600 MHz, DMSO-*d*_6_) spectral data are listed in Table 1; ESIMS: *m/z* 380, 382 [M + Na]^+^; HR-ESI-MS: *m*/*z* 380.9841, 382.9822 (calcd. for C_16_H_11_N_2_O_3_BrNa, 380.9851, 382.9830).

Metagenediindole C (**2**): colorless crystal; ${\left[\alpha \right]}_{D}^{29}$ 0.001° (*c* = 0.1, MeOH); IR (KBr) (ν_max_): 3392, 1652 cm^−1^. UV (MeOH) λ_max_ (logε): 261 (4.03), 224 (3.59) nm. ^13^C NMR (125 MHz, DMSO-*d*_6_) and ^1^H NMR (600 MHz, DMSO-*d*_6_) spectral data were listed in Table 2; ESIMS: *m/z* 380, 382 [M + Na]^+^; HR-ESI-MS: *m*/*z* 380.9845, 382.9824 (calcd. for C_16_H_11_N_2_O_3_BrNa, 380.9851, 382.9830).

### 3.4. Transposon Mutagenesis and Sequence Analysis

Random transposon mutations were generated with an EZ-Tn5™<KAN-2> insertion kit (Epicenter) in accordance with the manufacturer’s instructions. The 25D7 fosmid and transposon reaction mixture were incubated at 37 °C for 4 h. The reaction was terminated by adding 1 μL of stop solution. Then, the mixture was incubated at 70 °C for 10 min, electroporated into competent epi300 cells, and recovered in LAK (100 µg/mL ampicillin and 50 µg/mL kanamycin) plates. Colorless fosmid clones were selected randomly for sequencing (Sangon Inc., Shanghai, China).

### 3.5. Cytotoxic Activity

The cytotoxic activities of these compounds were evaluated using MCF7, B16, CNE2, Bel7402, and HT1080 cell lines by the CCK8 method [9]. Human breast adenocarcinoma cell MCF7, melanoma cell B16, nasopharyngeal cell CNE2, hepatoma cell BEL7402, and osteosarcoma cell HT1080 (CCTCC, Wuhan, China) were grown in Dulbecco’s modified Eagle medium) supplemented with 10% fetal bovine serum and 1% (*w/v*) penicillin/streptomycin and were seeded as 100 μL aliquots into a sterile 96-well microtiter plate at a titer of approximately 1000 cells per plate and incubated at 37 °C in 5% CO_2_ for 24 h. Compounds **1** and **2** resuspended in DMSO and a compound-free DMSO control were diluted in a fresh medium and added to the appropriate wells at final concentrations of 100, 50, 25, 13, 6.3, 3.1, 1.6, 0.78, 0.39, and 0.20 μg·mL^−1^. These plates were then cultured for another 72 h. A CCK8 assay was conducted to assess the cytotoxic effects of compounds **1** and **2** on the cells. In brief, 10 μL of CCK8 solution (Dojindo Laboratories, Kumamoto, Japan) was added to each well, and the 96-well plate was incubated at 37 °C for 2 h. The OD of each well was read at a wavelength of 450 nm to determine the cell survival rate by using a microplate reader (Epoch; Biotek, Winooski, VT, USA). The assay was repeated thrice. IC_50_ was calculated using Origin 7.5 (OriginLab, Northampton, MA, USA).

## 4. Conclusions

Metagenomics can be applied to investigate the secondary metabolites produced by large collections of bacteria present in extreme environments, such as deep sea, which remain recalcitrant to culturing. Metagenomics is also a major topic of international life science research. With modern molecular biology techniques, biosynthetic gene clusters with specific plasticity can be modified directly and expressed heterologously to create new structures called “artificial” compounds [16,17] with high activity and low toxicity. With these approaches, the production of microbial secondary metabolites can be improved to generate innovative drugs and to increase output. In our study, two new *bis*-indoles were obtained from 25D7 cultivated with 5-bromoindole, and their biosynthetic functional genes were determined. Our results revealed the heterologous expression and function of the 25D7 gene cluster.

## Figures and Tables

**Table 1:** ^1^H, ^13^C NMR data and ^1^H–^1^H COSY, HMBC correlations of compound **1**.

Position	δ_H_ (*J* in Hz)	δ_C_, Multiple		^1^H–^1^H COSY	HMBC
1-NH	10.58 br.s				n.o.
2		178.8	C		
3		77.8	C		
3a		137.2	C		
4	7.07 overlapped	127.7	CH	H-6	C-3, 5, 6, 7a
5		113.7	C		
6	7.40 dd (8.3, 1.5)	132	CH	H-4, 7	C-4, 5, 7a
7	6.90 d (8.2)	112.1	CH	H-6	C-3, 3a, 5
7a		141.8	C		
1′-NH	10.95 s			H-2′, 3′	C-2′, 3′, 3′a
2′	7.07 overlapped	124.8	CH	H-1′, 3′	C-3′, 3′a, 7′a
3′	5.91 br.s	101	CH	H-2′	C-2′, 3′a, 7′a
3′a		126.6	C		
4′		122.7	C		
5′	7.06 d (8.1)	117.4	CH	H-6′	C-2, 3, 3′a, 6′, 7′
6′	6.51 d (8.1)	104.8	CH	H-5′	C-4′, 7′, 7′a
7′		144	C		
7′a		126.3	C		
3-OH	6.58 br.s				C-2, 3, 3a, 4′
7′-OH	9.67 br.s				n.o.

n.o. is not observed.

**Table 2:** ^1^H, ^13^C NMR data, and ^1^H–^1^H COSY and HMBC correlations of compound **2**.

Position	δ_H_ (*J* in Hz)	δ_C_, Multiple		^1^H–^1^H COSY	HMBC
1-NH	10.49 s				n.o.
2		178.4	C		
3		75.4	C		
3a		136.5	C		
4	7.27 br.d (2.0)	127.8	CH	H-6	C-3, 5, 6, 7a
5		113.7	C		
6	7.42 dd (8.3, 2.0)	132	CH	H-4, 7	C-4, 5, 7a
7	6.87 d (8.3)	112.2	CH	H-6	C-3, 3a, 5
7a		141.4	C		
1′-NH	10.88 br. S			H-2′	C-2′, 3′, 3′a
2′	7.03 d (2.4)	123.4	CH	H-1′	C-3, 3′, 3′a, 7′a
3′		115.5	C		
3′a		126.9	C		
4′	6.69 d (7.5)	111.3	CH	H-5′, 6′	C-3′, 6′, 7′a
5′	6.66 t (7.5)	119.9	CH	H-4′, 6′	C-3′, 3′a, 6′, 7′
6′	6.45 d (7.3)	105.9	CH	H-4′, 5′	C-4′, 7′, 7′a
7′		144.1	C		
7′a		127.4	C		
3-OH	6.48 br.s				n.o.
7′-OH	9.57 br.s				n.o.

n.o. is not observed.

**Table 3:** Predicted hydroxylase genes of 25D7.

CDS No.	Predicted Gene	BLAST Result
20	phenol hydroxylase subunit	83aa, 89% identity to *Marinobacter algicola* DG893
21	phenol hydroxylase component phL	333aa, 85% identity to *Pseudomonas* sp. OX1
22	phenol hydroxylase component phM	89aa, 96% identity to *Pseudomonas* sp. OX1
23	phenol hydroxylase P3 protein	515aa, 94% identity to *M. algicola* DG893
24	phenol hydroxylase conserved region	119aa, 84% identity to *M. algicola* DG893
25	ferredoxin: oxidoreductase FAD/NAD(P)-binding	353aa, 96% identity to *M. algicola* DG893

## Supplementary Materials

The following are available online at www.mdpi.com/1660-3397/14/6/107/s1, Figure S1: Isolated compounds related to the biosynthetic pathway of 5-bromometagenediindole B/C, Figure S2: Compounds related to the biosynthetic pathway of 5-bromometagenediindole B/C, detected by HPLC-MS spectum.
