# Supplementary material for: Biosynthetic Functional Gene Analysis of Bis-Indole Metabolites from 25D7, a Clone Derived from a Deep-Sea Sediment Metagenomic Library
Source: Mar Drugs. 2016 Jun 1;14(6):107. doi: 10.3390/md14060107 (PMC4926066; doi:10.3390/md14060107)
Supplement: Supplementary file 1 [file marinedrugs-14-00107-s001.pdf]

Supplementary Materials: Biosynthetic Functional Gene Analysis of *Bis*-Indole Metabolites from 25D7, a Clone Derived from a Deep-Sea Sediment Metagenomic Library

Xia Yan, Xi-Xiang Tang, Dan Qin, Zhi-Wei Yi, Mei-Juan Fang, Zhen Wu and Ying-Kun Qiu

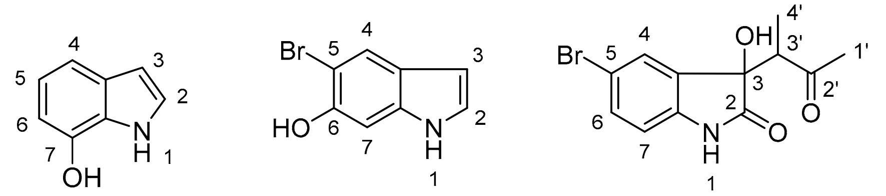

I **II** **III**

Figure S1. Isolated compounds related to the biosynthetic pathway of 5-bromometagenediindole B/C.

Spectra Data of Compounds I–IV

1*H*-Indol-7-ol (I)

A colorless crystalline solid; ESI-MS (*m*/*z*): 156 $[M + Na]^+$; ^{1}H-NMR (DMSO-d_6, 600 MHz) δ: 10.88 (1H, br.s, H-1), 9.53 (1H, br.s, 7-OH), 7.21 (1H, dd, *J* = 4.9, 2.5 Hz, H-2), 7.00 (1H, m, H-4), 6.79 (1H, m, H-5), 6.79 (1H, m, H-6), 6.36 (1H, m, H-3); ^{13}C-NMR (DMSO-d_6, 150 MHz) δ: 144.1 (C-7), 130.1 (C-3a), 126.5 (C-2), 125.0 (C-7a), 119.9 (C-5), 111.6 (C-4), 105.7 (C-6), 101.8 (C-3).

5-Bromo-1*H*-indol-6-ol (II)

A yellow amorphous solid; ESI-MS (*m*/*z*): 235 $[M + Na]^+$; ^{1}H-NMR (DMSO-d_6, 600 MHz):10.83 (1H, br.s, H-1), 9.68 (1H, br.s, 6-OH), 7.61 (1H, s, H-4), 7.16 (1H, t-like, *J* = 2.8, 2.6 Hz, H-2), 6.98 (1H, s, H-7), 6.26 (1H, t-like, *J* = 2.2, 2.0 Hz, H-3).

5-Bromo-3-hydroxy-3-(3-oxobutan-2-yl) indolin-2-one (III)

A yellow amorphous solid; ESI-MS (*m*/*z*): 320 $[M + Na]^+$; ^{1}H-NMR (DMSO-d_6, 600 MHz) δ: 10.42 (1H, s, H-1), 7.39 (1H, dd, *J* = 8.3, 8.3 Hz, H-6), 7.37 (1H, d, *J* = 2.2 Hz, H-4), 6.76 (1H, d, *J* = 8.1 Hz, H-7), 3.16 (1H, q, H-8), 2.10 (3H, s, H-10), 1.77 (3H, d, H-11).

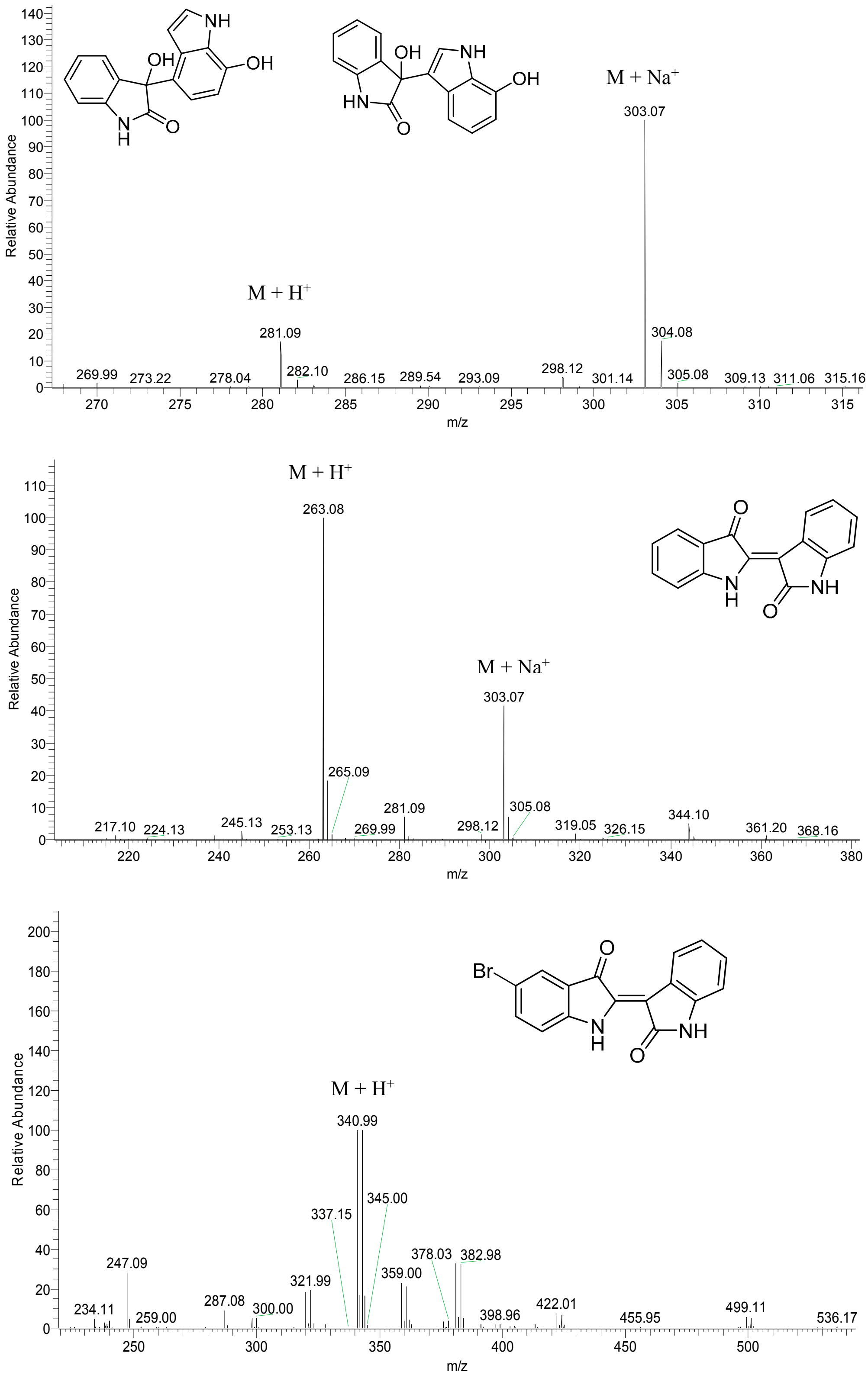

Figure S2. Compounds related to the biosynthetic pathway of 5-bromometagenediindole B/C, detected by HPLC-MS spectum.

© 2016 by the authors; licensee MDPI, Basel, Switzerland. This article is an open access article distributed under the terms and conditions of the Creative Commons Attribution (CC-BY) license (http://creativecommons.org/licenses/by/4.0/).
